# Short and long term effects of a lifestyle intervention for construction workers at risk for cardiovascular disease: a randomized controlled trial

**DOI:** 10.1186/1471-2458-11-836

**Published:** 2011-10-31

**Authors:** Iris F Groeneveld, Karin I Proper, Allard J van der Beek, Vincent H Hildebrandt, Willem van Mechelen

**Affiliations:** 1 Department of Public and Occupational Health, EMGO+ Institute for Health and Care Research, VU University Medical Center, Van der Boechorststraat 7, 1081 BT Amsterdam, The Netherlands; 2 Body@Work, Research Center Physical Activity, Work and Health, TNO-VUmc, Van der Boechorststraat 7, 1081 BT Amsterdam, The Netherlands; 3 TNO Quality of Life, Department of Physical Activity and Health, Wassenaarseweg 56, 2301 CE Leiden, The Netherlands

## Abstract

**Background:**

The prevalence of overweight and elevated cardiovascular disease (CVD) risk among workers in the construction industry is relatively high. Improving lifestyle lowers CVD risk and may have work-related benefits. The purpose of the study was to evaluate the effects on physical activity (PA), diet, and smoking of a lifestyle intervention consisting of individual counseling among male workers in the construction industry with an elevated risk of cardiovascular disease (CVD).

**Methods:**

In a randomized controlled trial including 816 male blue- and white-collar workers in the construction industry with an elevated risk of CVD, usual care was compared to a 6-month lifestyle intervention. The intervention consisted of individual counseling using motivational interviewing techniques, and was delivered by an occupational physician or occupational nurse. In three face to face and four telephone contacts, the participant's risk profile, personal determinants, and barriers for behavior change were discussed, and personal goals were set. Participants chose to aim at either diet and PA, or smoking. Data were collected at baseline and after six and 12 months, by means of a questionnaire. To analyse the data, linear and logistic regression analyses were performed.

**Results:**

The intervention had a statistically significant beneficial effect on snack intake (β-1.9, 95%CI -3.7; -0.02) and fruit intake (β 1.7, 95%CI 0.6; 2.9) at 6 months. The effect on snack intake was sustained until 12 months; 6 months after the intervention had ended (β -1.9, 95%CI -3.6; -0.2). The intervention effects on leisure time PA and metabolic equivalent-minutes were not statistically significant. The beneficial effect on smoking was statistically significant at 6 (OR smoking 0.3, 95%CI 0.1;0.7), but not at 12 months (OR 0.8, 95%CI 0.4; 1.6).

**Conclusions:**

Beneficial effects on smoking, fruit, and snack intake can be achieved by an individual-based lifestyle intervention among male construction workers with an elevated risk of CVD. Future research should be done on strategies to improve leisure time PA and on determinants of maintenance of changed behavior. Considering the rising prevalence of unhealthy lifestyle and CVD, especially in the aging population, implementation of this intervention in the occupational health care setting is recommended.

**Trial registration:**

Current Controlled Trials ISRCTN60545588

## Background

In the Netherlands, among men aged younger than 65 years, a quarter of all deaths is due to cardiovascular diseases (CVD) [1]. Cardiovacular disease may not only lead to premature death, but also to decreased physical functioning and lower quality of life [2]. Important precursors of CVD are obesity, hypertension, and an abnormal blood lipid profile. These abnormalities are caused to a large extent by an unhealthy lifestyle, such as unhealthy diet [3], insufficient physical activity (PA) [4], and smoking [5]. Irrespective of other CVD risk factors such as age, male gender, family history, or low job control [6], improving lifestyle will lower CVD risk. Effective lifestyle change strategies should be developed in order to prevent CVD.

Numerous trials have been performed among persons with an elevated CVD risk, assessing the effectiveness of interventions aimed at changing lifestyle [7-9]. Different strategies have been evaluated, among which providing advice, exercise classes, a prescribed diet, and individual counseling. Advice alone was proven to be less effective than individual counseling in achieving long-term behavior change among adults with an elevated CVD risk [8,9]. Supervised exercise and diet alone may facilitate body weight loss [10], but long-term behavior change is less likely if not combined with more intensive diet and PA modification therapy [11], such as individual counseling. Nowadays, a frequently used counseling method is motivational interviewing (MI). Motivational interviewing was originally developed for changing addictive behaviors, but has also proven effective in lifestyle change [12,13]. Motivational interviewing is a client-centered, directive method for enhancing intrinsic motivation to change by exploring and resolving ambivalence, and is based on the principle that behavior change occurs in stages [14]. During MI, determinants are addressed that have consistently proven associated with behavior change, such as attitude and self-efficacy [15].

The workplace is an appropriate setting for investigating lifestyle interventions, since many adults of various socio-economic statuses, lifestyles, and risk profiles can be targeted at once. Moreover, in the working population, a lifestyle intervention will not only influence CVD risk. Improving diet and increasing PA may also lower absenteeism [16]. Regular PA may increase work ability, due to its effects on cardiorespiratory and musculoskeletal capacity, factors that normally decline with age [17]. However, in most workplace lifestyle intervention studies, the sustenance of health effects, which is necessary for permanent CVD risk reduction, was not determined, as the final follow-up measurement took place directly after the intervention had ended [18,19]. Recently, two workplace intervention studies were performed on the effectiveness of MI on lifestyle changes [20,21], showing that MI is more effective than providing health risk information only, and equally effective to group activities or computer-tailored advice. Again, in both studies, no long-term follow-up measurements took place.

In the Health under Construction study, we aimed to develop and evaluate a lifestyle intervention for construction workers with an elevated CVD risk in the Netherlands. In this population, most workers are male and over 40 years of age. In 2008, the prevalence of overweight and obesity among male construction workers who attended a periodical health screening at the occupational health service was higher than in the total Dutch adult male population; 63.8% versus 52.3% [22]. According to the Framingham risk score [23], 27.3% of male construction workers had a higher than moderate 10-year risk of coronary heart disease. In the Health under Construction study, we evaluated the short- and long-term effects on PA, diet, and smoking of a lifestyle intervention consisting of individual counseling using MI techniques among male workers in the construction industry with an elevated risk of CVD in the Netherlands.

## Methods

### Participants

All male construction workers aged 18-65 years, employed at different (>400) companies throughout the Netherlands, who had attended the voluntary periodical health screening at the occupational health service between January 2007 and February 2008 (59.4% of all invited), and who had an elevated risk of CVD (n = 4.058; 19.1% of all screened), were personally invited to the study. Elevated CVD risk was concluded if the worker's 10-year coronary heart disease risk was higher than moderate according to the Framingham risk score, and he additionally fulfilled at least one of the following criteria; body mass index (BMI) ≥ 30 kg/m^2^; HbA1c ≥ 6.5%; consuming ≥ 35 glasses of alcohol per week; not meeting the Dutch PA guidelines; heart complaints; psychological complaints. The Medical Ethics Committee of the VU University Medical Center approved the study protocol. An extensive description of the study design is provided elsewhere [24].

### Randomization, blinding, and sample size calculation

The workers who consented to participate were pre-stratified for work type (blue-collar workers performing the construction work versus white-collar workers involved in administration and supervision), and individually randomized into the control or the intervention group, using Random Allocation Software (Version 1.0). After randomization, the research assistant notified each participant to which group he had been allocated. The investigator who performed the data analyses was blinded to the group allocation. Due to the study design, the intervention providers and participants could not be blinded. The sample size was based on PA; one of the main outcome measures of the study. To detect a 10% difference between the control and intervention group in the proportion of participants meeting none of both Dutch guidelines for moderate and vigorous intensity PA after 6 months, i.e. 38% in the control group and 28% in the intervention group, with a power of 80% and a 95% confidence interval (α = 0.05), 692 persons were needed at the first follow-up measurement.

### Intervention and control condition

Over a period of 6 months, each participant in the intervention group had three 45-60 minute face to face and four 15-30 minute telephone contacts with an occupational physician or occupational nurse. This counselor applied a client-centered counseling style using MI techniques such as asking open questions, summarizing, carefully listening, supporting, and raising ambivalence. In the first session, a stepwise protocol had to be followed. First, the participant's CVD risk profile was presented and his current health status was discussed. Second, the participant decided to aim at PA and diet, or smoking. Third, the participant was encouraged to indicate advantages and disadvantages of current and 'desired' behavior. Fourth, the participant was asked to indicate his willingness, readiness, and perceived confidence in his ability to change on 10-point scales. Last, the participant set long- and short-term goals, and formulated implementation intentions [25]. In the following counseling sessions, progress and barriers were discussed. The participants in the control group received usual care, consisting of brief oral or written information from the occupational physician about their risk profile, based on the periodical health screening results. To all participants of both intervention and control group, brochures were provided containing information on PA, healthy eating, smoking cessation, and CVD.

### Outcome measures

At baseline and after 6 and 12 months, a questionnaire on PA, diet, and smoking was filled out. For measuring PA, we used the fairly reliable (r_spearman _0.58) and valid (r_spearman _0.45) Short QUestionnaire to ASsess Health enhancing PA (SQUASH)[26]. By means of this questionnaire, we determined the number of minutes per week spent on two domains of PA, i.e. leisure time PA (walking, cycling, doing odd jobs, gardening), and sports activities. In addition, the total weekly amount of energy expended due to the activities in those two domains was estimated, by multiplying the total number of minutes spent on each activity by its metabolic equivalent- (MET-) value [27] and summing all MET-minutes. With regard to diet, in line with the Short Questionnaire for Measuring Fruit and Vegetable Intake, the average weekly intake during the past month was determined for fruit (pieces) and vegetables (heated and raw; tablespoons). Moreover, consumption of alcohol (glasses), and snacks were assessed. The latter food group was defined as the sum of sweet (e.g. piece of pie), cold salty (e.g. handful of crisps), and warm salty snacks (e.g. piece of egg roll), eaten outside the regular meals. The food questionnaire was not validated but tested for face validity by an expert in nutrition and lifestyle change, and for comprehensibility by two construction workers. Current smoking status was defined as 'smoker' or 'non-smoker'. Furthermore, the participant was asked whether he had used nicotine replacement therapy or medication in case he had succeeded in smoking cessation. At baseline, the possible confounding variables age (years) and BMI were determined at the occupational health service. For determining BMI, body height (meters) without shoes was determined with the participant in standing position, his heels and head against the wall and his face in a horizontal plane. Body weight (kilograms) was measured without shoes and jacket, using a digital balance.

### Data analyses

Data were analyzed using SPSS (Version 15.0, Chicago Ill). In the baseline questionnaire, all participants had indicated whether they would prefer to improve their dietary or PA behavior, or to quit smoking. Those who had indicated to prefer improving diet or PA (energy balance-related behaviors; EB) were analyzed separately from the ones who had indicated to prefer smoking cessation (SC). As a result of self selection, baseline differences between the intervention and control group may have arisen, possibly leading to confounding. Therefore, we checked for baseline differences between intervention and control group in age, BMI, smoking status and all of the outcome measures, within both the EB group and the SC group. To determine the effects at 6 months, linear and logistic regression analyses were done with the variable of interest as the outcome, and group allocation (intervention vs. control) as the independent variable, adjusted for the baseline value. The effects at 12 months were evaluated using the same method. In both the EB and SC group we checked for confounding by age and BMI, and for effect modification by variables that theoretically could modify the effect on the outcome measure of interest, i.e. age, BMI, smoking, work type, and marital status (partner/no partner). Effect modification was concluded in case the p-value of the interaction term was <0.1. Only participants for whom data were present on all three time points were included in the analyses. Additionally, in order to assess the intervention effects among participants who had adhered to the protocol, linear and logistic regression analyses according to the 'per protocol' principle were done, for the effects at 6 and at 12 months. Of the intervention group, only those who had completed five or more counseling sessions on one of both topics (diet and PA, or smoking cessation) were included in the analyses. Of the control group, only those participants were included who had indicated *not *to have received counseling sessions and/or tailored lifestyle advice from any type of care provider, either face to face, by telephone, or computerized, between baseline and 6 months.

## Results

### Baseline characteristics and confounding

**Figure 1:**
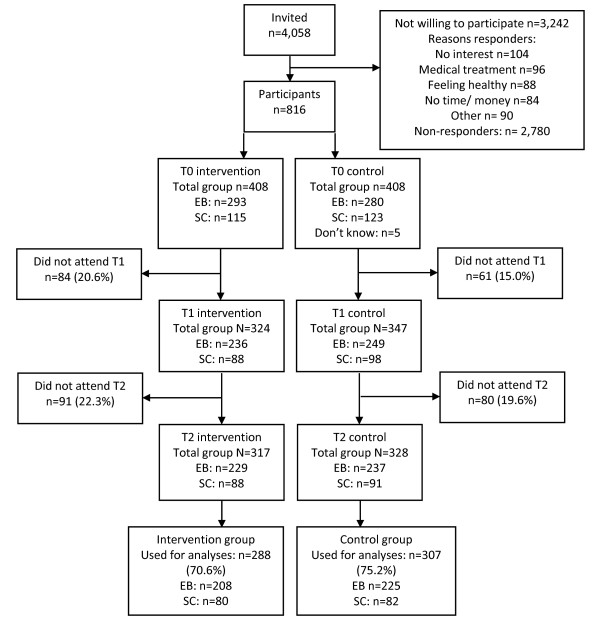
**Flow diagram of the progress through the phases of the study**.

In Figure 1 the participant flow is presented. 288 Participants in the intervention group and 307 participants in the control group were included in the analyses. At baseline, in the EB group, 31.1% was a blue-collar worker and 69.9% was a white-collar worker, mean age was 47.4 years (standard deviation [SD] 8.8), mean BMI was 28.8 (SD 3.5), and 32.7% was a current smoker. BMI appeared to be a confounder for snack intake. For consistency reasons, all analyses in the EB group were adjusted for BMI. In the SC group, at baseline, 85.2% was a blue-collar worker and 14.8% was a white-collar worker, mean age and mean BMI were 46.8 (SD 8.9) and 27.8 (SD 3.2), respectively, and 100% was a current smoker. In the SC group, no confounding by age or BMI was concluded, thus no adjustments were made. No adverse events of the intervention were reported by any of the participants.

### Physical activity

In Table 1, the values for leisure time PA, sports, and MET-minutes at baseline, 6 and 12 months, and the results of the linear regression analyses are presented, for the EB group only. No statistically significant intervention effects were found for any of those variables at 6 or 12 months, although the improvements in leisure time PA, sports, and MET-minutes at six months were largest in the intervention group. BMI was an effect modifier for leisure time PA at 6 months. The normal weight participants (BMI<25; n = 52) in the intervention group decreased the time spent on leisure time PA (β -133.3, 95%CI -330.7; 64.1), whereas the overweight (BMI≥25 and <30; n = 221) and obese (BMI≥30; n = 156) participants increased their leisure time PA (β 68.9, 95%CI -29.6; 167.4 and β 104.6, 95%CI -15.7; 224.9). Age appeared to modify the 6- and 12-month effects of the intervention on leisure time PA as well. The intervention effect among participants aged 45 years and over (n = 254) was larger than among the younger ones (n = 175) at both 6 (β 88.8, 95%CI -10.1; 187.7 vs. β 21.0, 95%CI -76.6; 118.6) and 12 months (β 86.1, 95%CI -19.3; 191.5 vs. β -53.5, 95%CI -156.9; 49.9). In none of the BMI and age subgroups, statistically significant intervention effects were found.

**Table 1:** Intervention effects in the energy balance subgroup, as determined by linear regression analysis, adjusted for body mass index.

	n	Baseline mean (SD)	6 months mean (SD)	β 6 months (95%CI)	12 months mean (SD)	β 12 months (95%CI)
**Leisure time physical activity (minutes per week)**						
Intervention	207	429.7 (390.0)	589.7 (464.2)	59.5 (-11.3; 130.3)	543.4 (462.5)	30.2 (-45.3; 105.8)
Control	222	466.0 (349.0)	552.8 (424.6)		529.4 (409.2)	
**Sports activities (minutes per week)**						
Intervention	207	95.1 (116.6)	107.3 (130.7)	10.1 (-9.6; 29.7)	109.9 (139.8)	2.2 (-19.0; 23.5)
Control	223	77.9 (126.8)	84.7 (139.0)		96.5 (143.3)	
**Leisure time physical activity- and sports-related energy expenditure (MET-minutes per week) **						
Intervention	206	2,130.5 (1,494.9)	2,852.4 (1,769.7)	226.5 (-81.6;534.5)	2,678.6 (1,838.4)	132.2 (-177.7; 442.0)
Control	223	2,209.5 (1,543.8)	2,672.5 (2,060.1)		2,591.1 (1,899.0)	
**Alcohol (glasses per week)**						
Intervention	196	9.6 (9.9)	8.8 (9.07)	-1.3 (-2.7; 0.1)	8.8 (8.9)	-1.0 (-2.5; 0.4)
Control	213	9.6 (9.5)	10.2 (10.21)		9.9 (10.6)	
**Snacks (pieces per week)**						
Intervention	207	14.1 (11.21)	11.1 (11.1)	-1.9 (-3.7; -0.02)*	12.1 (9.2)	-1.9 (-3.6; -0.2)*
Control	220	13.1 (9.8)	12.6 (11.9)		13.6 (11.2)	
**Fruit (pieces per week)**						
Intervention	207	10.1 (6.9)	11.8 (8.1)	1.7 (0.6; 2.9)*	11.7 (8.3)	0.9 (-0.2; 2.1)
Control	221	10.8 (8.6)	10.7 (7.9)		11.3 (7.8)	
**Vegetables (spoons per week)**						
Intervention	207	17.0 (9.5)	18.0 (9.2)	0.9 (-0.6; 2.4)	17.5 (8.8)	0.04 (-1.4; 1.4)
Control	222	17.7 (10.3)	17.3 (8.3)		7.7 (8.8)	

. * p < 0.05

SD: standard deviation; CI: confidence interval; MET: metabolic equivalent.

### Diet

In Table 1, the values for fruit, vegetables, alcohol and snacks at baseline, 6, and 12 months, and the results of the linear regression analyses are presented, for the EB group only. At 6 months, a statistically significant beneficial intervention effect was found for snack and fruit intake, and a borderline significant effect was found for alcohol. At 12 months, the intervention effect on snack intake was still statistically significant. Due to effect modification by age, the intervention effect appeared statistically significant among participants aged 18-44 (β -3.2, 95%CI -5.9; -0.5) only, but not among those aged 45 and over (β -1.1, 95%CI -3.2; 1.1). At 12 months, the effect on alcohol intake was modified by BMI. The normal weight participants significantly lowered their alcohol intake as a result of the intervention (β -3.8, 95%CI -7.6; -0.04), whereas the overweight and obese did not (β -0.8, 95%CI -2.9; 1.3 and β -0.4, 95%CI -2.7; 1.9).

### Smoking

In Table 2, the results of the logistic regression analyses in the SC group can be found. At 6 months, 25 persons (31.3%) in the intervention group had quit smoking, as opposed to 11 (13.4%) in the control group. The odds ratio (OR) of smoking was 0.3 (95%CI 0.1; 0.7). Nine (36%) persons in the intervention group who quit smoking had used nicotine replacement therapy or medication, as opposed to 2 (18%) in the control group. The statistically significant effect was not sustained until 12 months follow-up (OR 0.8, 95%CI 0.4; 1.6). Age appeared a modifier of the intervention effect on smoking. The intervention was more effective among participants aged 45 years and over (n = 100) than for the younger ones (n = 62) at both 6 (OR 0.1, 95%CI 0.02; 0.5 vs. OR 0.7, 95%CI 0.2; 2.1) and 12 months (OR 0.4, 95%CI 0.1; 1.2 vs. OR 1.2, 95%CI 0.4; 3.8).

**Table 2:** Intervention effects in the smoking cessation subgroup, as determined by logistic regression analysis.

	n	Baseline %	6 months %	OR (95% CI)	12 months %	OR (95% CI)
**Smoking (%)**						
Intervention	80	100	68.7	0.3 (0.1; 0.7)*	76.3	0.8 (0.4; 1.6)
Control	82	100	86.6		80.5	

* p < 0.05

OR: odds ratio; CI: confidence interval.

### Per protocol analyses

According to the per protocol analyses, the differences between control and intervention group at both 6 and 12 months were larger than in the analyses of the study population as a whole. Again, the effect on fruit intake was statistically significant at 6 months (β 0.9, 95%CI 0.7; 3.1). Also, the effect on alcohol intake was statistically significant at 6 months (β -1.7, 95%CI -3.2; -0.1). The effect on snack intake was statistically significant at 6 months (β -2.6, 95%CI -4.5; -0.6) and at 12 months (β -3.2, 95%CI -5.0; -1.5). The effect on smoking was statistically significant at 6 months (OR 0.1, 95%CI 0.05; 0.3) as well as at 12 months (OR 0.4, 95%CI 0.2; 0.99).

## Discussion

### Findings

The aim of the study was to evaluate the effectiveness of a lifestyle intervention consisting of individual counseling using MI techniques for workers in the construction industry with an elevated risk of CVD. At 6 months, the intervention had a statistically significant beneficial effect on snack intake, fruit intake, and smoking. At 12 months, most of the initial improvements in the intervention group were still present, although the effects on smoking and fruit intake were no longer significant. At 12 months, i.e. 6 months after the intervention had ended, a significant effect was found on snack intake. Moreover, at 12 months, a statistically significant beneficial effect was found on alcohol consumption among the normal weight participants. No statistically significant intervention effects were found for time spent on leisure time PA and sports, or on MET-minutes per week. Work type did not modify any intervention effect, implying that the intervention is equally effective in blue- and white-collar workers.

### Interpretation of findings

Physical activity had substantially increased in both the intervention and the control group at 6 months (+172 vs. + 94 minutes per week) and at 12 months (+129 vs. + 84 minutes per week), but the intervention effect was not significant. This is not in line with the reviews of Dugdill et al. (2008) and Conn et al. (2009), who concluded evidence for the effect of workplace counseling on PA [28,29]. The lack of a significant intervention effect in our study may be related to the high levels of baseline PA at work, or due to the relatively large amount of time spent on doing 'odd jobs' outside working hours. The age difference in the increase in PA may be explained by the fact that younger workers spent significantly more time in sports at baseline than the older ones (111 minutes vs. 69 minutes per week). Participating in sports might have lowered their urge to undertake physical activities of moderate intensity in leisure time. The lack of a statistically significant effect on PA is unfortunate, since Ruzic et al. (2003) demonstrated that a high physical load at the workplace (or while doing odd jobs) did not induce positive changes in aerobic capacity, strength, or flexibility of male workers aged 20-60 [30]. Thus, in a future study, another strategy should be sought for stimulating engagement in leisure time PA among workers in the construction industry. With respect to diet, notable improvements were found in the intervention group. Pignone et al. (2003) also concluded that medium to high intensive interventions generally produced medium to large changes in dietary behavior, but they found no specific differences in effectiveness on fat or fruit and vegetable intake [31]. In our study, especially the consumption of snacks was affected, possibly because participants realized that decreasing snack intake has a direct effect on losing body weight, which may have been their main goal. The effect on snack intake was modified by age, implying a more favorable effect among the younger participants. Nonetheless, the difference in change was only small (-1.9 vs. -1.6 snacks per week), and the higher snack intake by the younger participants at baseline (+0.7) should be noted. Still, whereas younger participants were more likely to change snacking behavior, the older participants might have decided to change other aspects of diet, e.g. meal size, instead. Finding a significant and equally large effect 6 months after the intervention has ended is important, since only long-term changes in behavior will lead to a sustained decrease of CVD risk. The sustained effect on snack intake probably contributed to the statistically significant decrease in body weight of nearly 2 kg after 6 and 12 months, that we described in an earlier publication [32]. Also, the statistically significant improvement in high density lipoprotein (HDL-) cholesterol at 12 months that we found [32] might partly have been caused by the changes in dietary behavior. The mediating effect of snack intake in the changes in body weight and cholesterol remains to be determined. As to smoking, according to a recent Cochrane review, previous workplace individual counseling interventions aimed at smoking cessation resulted in quit rates between 6 and 21% in the intervention group [33]. As compared to those studies, the short-term quit rate in the intervention group in our study was high. The relatively high frequency and long duration of contacts of the intervention in our study may have contributed to this result. Another factor that may have contributed to the high cessation rate was the fact that most counselors provided information on nicotine replacement therapy to those clients who were motivated to quit. A combination of counseling and medication use increases success rates, as stated by Fiore et al. [34].

### Issues related to behavior change

Initial positive results at the short term and weakening of the effects at the longer term is a well-known phenomenon. Habitual behavior and convenience of the 'original' behavior may add to this effect. From this study we can conclude that it is vital to find out the determinants of *maintenance *of 'new' lifestyle behavior. As acknowledged by Pritchett et al. (2005), lifestyle modification often results in health improvements, but the challenge is to find out how to avoid relapse to old habits [35]. If we know why some people maintain new behavior and others do not, tools for prevention of relapse may be developed. Possibly, short three-monthly follow-up counseling sessions, either face to face or by telephone, may facilitate maintenance of behavior. As shown in the Finnish Diabetes Prevention Study, this strategy induced behavior change that was sustained at two years [9]. Another issue which may be targeted in future research is the possible additive effect on environmental changes. During our intervention, behavioral determinants were discussed that could be changed by the participant himself, such as attitude and self-efficacy. However, behavior change is not only determined by personal factors, but also by the environment [36]. When creating an environment in which the healthy choice is stimulated, such as healthy foods in the company restaurant, or the opportunity to visit a company fitness center [37], behavior change might be facilitated. Environmental changes are difficult to implement for blue-collar workers, who are physically active at work and usually work at different locations, but could be tested among white-collar workers in the construction industry.

### Changes in the control group

Not only the intervention group improved certain lifestyle behaviors, the participants in the control group did so as well. This is not surprising, since they had just received the results of their periodical health screening and were notified of being at risk for CVD. The 'measurement effect', as described by Van Sluijs et al. (2006) may also have been causal to the improvements in the control group found after 6 and 12 months [38]. Moreover, the 'Hawthorne' effect, i.e. altering behavior because one is aware of being part of an experiment, may have played a role. The substantial increase in PA in the control group may partly have resulted from the brochures they received at baseline. Last, one should keep in mind that persons who are intrinsically motivated to change lifestyle will be more likely to participate in a lifestyle intervention trial than those who are not. Since these mechanisms apply to both the intervention and the control group, the intervention effects have probably not been distorted.

### Limitations and strengths

Some limitations of this study should be mentioned. A well-known limitation is over- and underreporting of behavior. Since this 'misreporting' occurred in both groups, it has probably not introduced bias, although it may have attenuated the intervention effects. Another drawback is the fact that no validated questionnaire was used for measuring dietary intake. We had two reasons for doing so. First, we wanted the questionnaire to be completed by all participants, and considered the validated food frequency questionnaires too extensive for this purpose. Second, we aimed to determine the intervention effects on certain health-related food groups, and not on exact intake of grams or energy. Another limitation is the fact that participants were not blinded. In our study, the chance of contamination between groups was limited, since the workers were employed at more than 400 different companies, and recruited and randomized individually. A limitation related to the study population may be selective participation and dropout. The participants were older and more likely to smoke than those who did not participate, and the drop-outs were younger and less likely to smoke than the participants [39]. The differences between the target group and the study completers may slightly lower the generalizability of the results. Nevertheless, the age- and lifestyle-related characteristics of the drop-outs were equal in the intervention and the control group. Last, by analyzing the EB and SC group separately, the study may have become underpowered. Since differences between intervention and control group were checked and adjusted for, both within the EB and SC groups, no confounding will have occurred. This study also has numerous strengths. Compliance to the intervention was rather high, i.e. two-thirds of participants in the intervention group had five or more counseling sessions [40]. Only few participants in the control group had received lifestyle advice from another care provider, thus the contrast between groups was large. Randomization was performed at the individual level, which is the preferred method since baseline differences between intervention and control group are least likely. With respect to the analyses, the participants in the EB group were analyzed separately from those in the SC group, in order to determine changes in the lifestyle behaviors that were actually aimed at. Furthermore, the study can be considered as an effectiveness study as opposed to an efficacy study. Namely, counseling was conducted at the occupational health service and performed by an occupational health service professional instead of by the researchers themselves. As the results of this study reflect the intervention effectiveness in 'real life', decision makers will better be able to decide upon implementation. The most important strength is that we investigated the effects on CVD risk-related behaviors no less than 6 months after the intervention had ended. With this study, we generated knowledge on the effectiveness of a promising counseling strategy on behavior change among a population in which CVD risk will be rising in the following years.

## Conclusions

We conclude that this lifestyle intervention for workers in the construction industry at risk for CVD had significant effects on snack and fruit intake and smoking at 6 months. The significant effects on snack intake were unchanged at the long term. Future studies should be done on strategies for changing leisure time PA, and on determinants of maintenance of changed behavior in this population. Considering the rising prevalence of unhealthy lifestyle and CVD, especially in the aging population, implementation of this intervention in the occupational health care setting is recommended.

## Competing interests Statement

The authors declare that they have no competing interests.

## Authors' contributions

IG performed the analyses and wrote the manuscript. KP wrote the original study protocol. KP, AB, VH and WM provided intellectual input and had a role in supervision. All authors read and approved the final manuscript.

## Pre-publication history

The pre-publication history for this paper can be accessed here:

http://www.biomedcentral.com/1471-2458/11/836/prepub
